# Analysis of Permeation and Diffusion Coefficients to Infer Aging Attributes in Polymers Subjected to Supercritical CO_2_ and H_2_ Gas at High Pressures

**DOI:** 10.3390/polym14183741

**Published:** 2022-09-07

**Authors:** Hamad Raheem, Bernadette Craster, Ashwin Seshia

**Affiliations:** 1 Department of Engineering, University of Cambridge, Trumpington St., Cambridge CB2 1PZ, UK; 2 Materials and Structural Integrity, TWI Ltd., Granta Park, Cambridge CB21 6AL, UK

**Keywords:** HDPE, PE-RT, polyethylene, permeation, diffusion, solubility, supercritical, CO_2_, aging, PVDF, hydrogen

## Abstract

There is a need to understand the permeation flux behavior of polymers exposed to high-pressure and -temperature fluids continuously for long time intervals. This study investigates evidence of structural alterations in polymer specimens as indicators of material aging through the monitoring of transport coefficients at pressure steps from 10 barg to 400 barg and temperatures ranging between 30 °C and 90 °C. The continuous flow permeation methodology is a well-established technique described in the literature for applications from membrane separation processes to polymeric pressure barriers used for complex fluid containment in the oil and gas industry. In this study, a novel methodology has been used that allows the permeating flux of supercritical CO_2_ and H_2_ gas through raised-temperature polyethylene and polyvinylidene fluoride films at varying elevated temperatures and pressures to be determined, over timescales of several months using gas chromatography. During these long-term measurements, changes in the test conditions, principally in temperature and stepwise increases in differential gas pressure, were made in order to determine the activation energy for permeation along with the transport coefficients of permeation, diffusion, and sorption. At no time was the polymer film allowed to outgas during the temperature or pressure alterations. The permeation experiments are complemented by differential scanning calorimetry tests to track changes in polymer crystallinity before and after exposure of the specimen to plasticizing gases, which revealed the extent of structural alterations inflicted on the specimen due to high temperature and pressure loads. It is seen that specimens that were exposed to starting high pressures aged more than those that had gradual increases in feed pressure. Furthermore, the relationship between transport coefficients and fractional free volume in the polymer upon exposure to high pressure and temperature conditions is explored. Lastly, the benefit of using fugacity in place of feed pressure for the calculation of the permeability coefficient is discussed. This study contributes to the understanding of the effect of prolonged exposure of the polymeric specimens to CO_2_ and H_2_ gas under stepwise pressure and temperature loading on their flux behaviors and crystallinity, and to candidate polyethylene-based specimens for oil field deployment.

## 1. Introduction 

Tracking the aging of polymeric materials that are in continuous exposure to supercritical CO_2_ is of interest in many industries as it allows not only an advance warning of potential failures in such systems but also an understanding of the long-term effects of this plasticizing agent on polymer specimens [1,2,3,4,5]. Transport through membranes can be quantified through three main methods [6,7,8]: (a) sorption–desorption, where a thin polymer film is immersed into a permeant solution and the mass difference of the film is recorded; (b) isochoric diffusion, where permeants diffuse from a donor compartment to a receiver compartment of constant volume, where they are detected; and lastly (c) isobaric diffusion, which encompasses of a constant pressure flowing stream permeating through a membrane and being detected in the receiver end via mass spectrometry or gas chromatography. The last method is often referred to as the continuous flow method and was chosen as the permeation experiment for this study because it allows studying the steady flux behavior of permeants as a function of feed pressure for prolonged time durations [9]. In addition, it is also used to understand the transport behavior of gases, especially gas mixtures [10,11], through membranes, and it was applicable accordingly to test the effect of using fugacity to determine transport coefficients against feed pressure.

Craster and Jones [9] conducted continuous flow permeation experiments to study the permeability of supercritical CO_2_ in the presence of 1.5% H_2_S through molded polyphenylene sulfide (PPS) films for temperatures and pressures up to 100 °C and 400 barg. The stepwise feed pressures were allowed to achieve steady-state fluxes. The experiments in this study build upon the methodology of Craster and Jones through the use of prolonged isobaric steps until the detected penetrates’ volumetric flux plateaus. The novelty in our methodology is the replication of continuous flow permeation with varying starting pressure steps that build up to 400 barg and applying this technique to polyethylene of raised temperature resistance (PE-RT) for the first time using supercritical CO_2_ and H_2_ gas. This is described in more detail in the methodology section.

Studies aiming to correlate the rate of supercritical CO_2_ permeation to crystallinity and structural attributes of polymers are widely reported in the literature [12,13,14,15,16,17]. For instance, the permeability coefficient in amorphous polyether ether ketone (PEEK) thin films was reported by McKeen [18] to be twice as large compared with a crystalline sample, which is supported by the findings of Cowling and Park [19] for H_2_, N_2_ and CO_2_ permeation through polybutadiene specimens. Heilman et al. [20] measured the permeability coefficient to be 4.3 × 10^−8^ cm^2^ s^−1^ bar^−1^ for polyethylene (PE) in the temperature range of 30–45 °C. Flaconneche et al. [21] determined transport coefficients of CH_4_-CO_2_ gas mixtures through PE and polyvinylidene at pressures up to 100 bar. Other studies on CO_2_ permeation to PE include those of Yasuda et al. [22], Yasuda and Peterlin [23], Sha and Harrison [24], Boyer et al. [25], and Sarassin et al. [26], which showed the effect of highly pressurized CO_2_ and CH_4_ on the transport coefficients and fractional free volume of high-density polyethylene (HDPE) via a molecular simulation approach. 

Similarly, the dependence of the transport coefficients on temperature and pressure changes was investigated, and it was shown that the permeability of a polymer to a gas may increase, remain constant, or decrease with feed conditions [7,15,16,17,27]. Kumazawa et al. [28] observed changes in the slope of permeability versus pressure Arrhenius plot at temperatures near the glass transition, $T_{g}$ of the specimen. This finding is supported by the Yampolskii et al. [29] observation of discontinuity in Ar and CO_2_ solubility coefficients through poly(trimethylsilyl norbornene) near its $T_{g}$ and by the finding of Toi et al. [30] showing changes in the poly(vinyl acetate) diffusion slope to Ar near its $T_{g}$. Furthermore, Rowe et al. [31] and Bernardo et al. [32] showed that the transport coefficients of polymers can be altered as they age. The aging can be translated as the removal of plasticizers or alterations in the internal arrangements of crystalline and amorphous phases. The latter is often obvious from the slope of the plateau on the permeation trace if the test is continued for a long enough period [9]. However, a literature search revealed the study of Craster and Jones [9] as the only published work for the empirical use of long-term continuous permeation tests to monitor ageing in polymers at elevated pressures and temperatures. This study aims to address an understanding of the substructure of PE-RT further with respect to induced aging through gas permeation, monitoring its aging behavior upon exposure to plasticizing transport fluids, and comparing it to conventional HDPE samples exposed to similar environments from the literature. This work is intended to inform subsequent studies, including those seeking to develop non-destructive means of assessing the alteration in the material structure inflicted by lengthy exposure to supercritical CO_2_.

## 2. Materials and Methods

In continuous flow permeation, CO_2_ gas is pressurized at one face of a polymer film that is supported by a porous steel frit, and nitrogen sweep of a known flow rate carries the permeated gases on the other side of the polymer film to a gas chromatograph (GC) [9]. Details of the experimental setup are presented in the methodology section. 

HDPE has a semicrystalline structure with the crystalline phase dominating at least 60% of the volume. The irregular and random distribution of the amorphous phase results in free volume within the structure that allows gases to permeate relatively quickly [9]. The rate of permeation, consequentially, is significantly influenced by the internal structure of polymers [13,17]. 

PE-RT has been an established ISO class of PE materials for domestic hot and cold water and industrial pipe applications for more than 20 years [33]. Having a unique crystalline microstructure that offers long-term hydrostatic strength at high temperatures without the need for chemical modification, post-extrusion curing, or cross-linking makes it an attractive product for high-temperature applications where conventional HDPE is restricted by temperature limitations. The bond-making process between chains gives the cross-linked material increased molecular weight and rigidity [34]. However, the process of cross-linking HDPE imposes additional manufacturing costs and time [33]. Given that PE-RT is manufacturable on a conventional HDPE extruder, it allows extending the use of PE-based pipes to higher-temperature industrial applications inexpensively. 

The method of continuous permeation utilized in this study is an advancement to the procedure conducted by Craster and Jones [9], who exposed polymer specimens to continuous permeation at stepwise pressure increases. However, to further explore the effect of high-pressure steps on transport coefficients, starting from those high pressures seemed necessary, especially for the diffusion coefficients determined through the time-lag technique. Consequentially, this study conducted two permeation rounds, where the first one used four permeation cells for identical pressure steps to validate transport coefficient calculations at a constant 60 °C and the following pressure steps: 10, 50, 100, 200, and 400 barg. The latter permeation run aimed to validate transport coefficients at higher pressures, in which 3 identical permeation cells were run simultaneously with different starting pressures leading to 400 barg, while all remaining variables were kept constant. The 1st cell included pressure steps 50, 100, 200, and 400 barg, while the 2nd started with 100 barg and the 3rd started with 200 barg, and all went in steps to 400 barg at a constant 60 °C. Moreover, in order to understand aging behavior with temperature, the pressure was then kept at 400 barg, and the temperature was varied from 30 °C to 90 °C while allowing the flux to reach a steady state at every new temperature.

All tests were conducted in TWI headquarters near Cambridge, UK. The laboratory has a central air monitoring system that provides an alarm when low levels of toxic gases are detected. The supply of gas mixtures comes from gas cylinders, controlled through bespoke valves and operated by highly skilled and trained staff. The valves close if the central safety system detects a gas leak in the laboratory.

**Figure 1:**
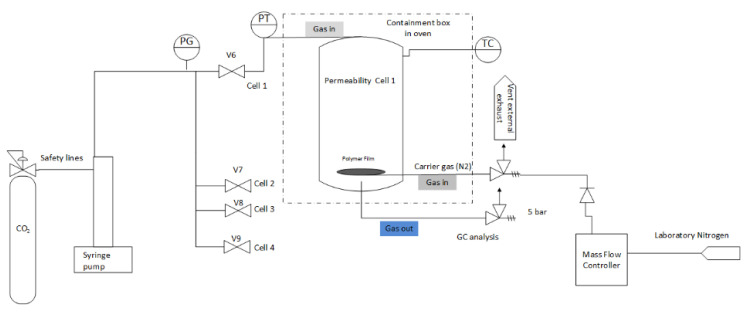
Custom-built rig used to expose polymers to supercritical CO_2_ mixtures at pressures up to 689 barg. PG is pressure gauge and PT is temperature gauge.

A schematic of the experimental rig used for the continuous permeation of supercritical CO_2_ is given in Figure 1. In this rig, a Teledyne syringe pump pressurizes gaseous mixtures to a maximum of 689 barg (9990 psi), measured through the integrated pressure transducer. Note that pressures are reported in bar gauge (barg) because the system is not placed under vacuum.

The gas, once pressurized, is supplied to four bespoke permeation cells housed in an oven from Binder GmBH operating in the temperature range of −10 to 300 °C. Each permeation cell contains a polymer disc and is supported by a stainless-steel sinter treated with SilcoNert coating provided by Silco Tek. The stainless-steel sinter is continuously purged by nitrogen gas at 10 mL/min, which sweeps the permeating gases to be analyzed by a GC. The GC was supplied by PerkinElmer, and further modifications were carried out by the facility for analysis. The described permeation method is commonly known as the continuous flow permeation measurement method [9,35].

The materials used in the study include a PE-RT specimen cut to 40 mm × 2 mm diameter discs, a polyvinylidene fluoride (PVDF) specimen cut to 40 mm by 2 mm diameter discs, and 99.99% CO_2_ gas sourced in cylinders from CK Special Gases Ltd.

For differential scanning calorimetry (DSC) measurements of PE-RT, which has a melting point of around 130 °C, samples were heated from 5℃ to 160 °C at a rate of 10 °C/min by a Perkin Elmer 4000. Then, samples were cooled to 5 °C and heated a second time to 160 °C at the same rate to determine the permanent effects of aging on the polymer structure. The small samples (~17 mg) were kept for 5 min at each temperature to eliminate any thermal history.

## 3. Background Theory

### 3.1. Classical Permeation—Solution-Diffusion Model

Analysis of gas mixture transport through a membrane requires a reliable experimental method that allows quantifying individual transported components. The continuous flow method is used to measure the transport of components of gas mixtures through membranes [10,11]. The permeability $K_{i}$ for species *i* is calculated using

(1) $$K_{i}=\frac{Q_{max,i}\;l}{A\Delta P_{i}\;},$$

where $Q_{max,i}$ is the maximum volume flow rate (cm^3^ (STP) s^−1^), *l* is the sample thickness (cm), *A* is the surface area (cm^2^) through which permeation occurs, and $\Delta P_{i}$ (barg) is the difference in the partial pressure of species *i* in the initial gas mixture. The partial pressure term is often replaced by the fugacity term to reflect the nonideality of the test fluid [36,37,38] and can be calculated using NIST REFPROP software. 

Furthermore, permeability (cm^3^ (STP) cm^−1^ s^−1^ bar^−1^) of a dense polymer depends on transient time, which can be expressed in terms of the diffusion coefficient (cm^2^ s^−1^), and affinity for the internal surface, which can be expressed in terms of the solubility coefficient (barg^−1^). The relationship is simply described by [39]:

(2) $$K_{i}=D_{i}S_{i}$$

In most cases, Henry’s law is assumed to hold, namely

(3) $$C_{H}=P_{i}S_{i},\;$$

where $C_{H}$ is the equilibrium gas concentration dissolved in the polymer membrane (g of gas per g of polymer), and it varies linearly with its partial pressure, $P_{i}$ [11]. Again, the partial pressure term can be replaced by the fugacity term [38].

A solubility prediction model was proposed by Sarrasin et al. [26] to describe sorption isotherms of the gas *i*:

(4) $$S_{i}=K_{Di}e^{\left(\sigma_{i}C_{i}+\beta P\right)}$$

where $K_{Di}$ is the Henry constant, $\sigma_{i}$ is described as the plasticization coefficient related to the sorbed gas concentration $C_{i}$, and β represents the hydrostatic effect produced by the gas pressure *P* applied to the polymer specimen.

### 3.2. Diffusion Coefficient by Time-Lag Method

For materials where the permeation process does not vary with the concentration of dissolved species, which is assumed to be the case for polymeric membranes, then the diffusion coefficient $D_{i}$, for species *i*, can be quantified using the time-lag method [11,40,41,42] as follows:

(5) $$Q_{acc,i}=\frac{D_{i}C_{i}}{l}\left(t-\frac{l^{2}}{6D_{i}}\right)\;$$

where $Q_{acc,i}$ is the cumulative mass per unit area of species *i* that has diffused through the polymeric membrane, which can be calculated by integrating the flux (*Q*) measured at the GC as a function of time; and $C_{i}$, which is the outer boundary concentration between inlet chamber and membrane. $D_{i}$, on the other hand, can be calculated from:

(6) $$D_{i}=\frac{l^{2}}{6\theta }\;$$

where θ is the time intercept in a plot of the accumulated volume of the permeant, $Q_{acc,i}$, versus time, also known as the time lag [9].

### 3.3. Diffusion Coefficient by Slope-Concentration Method

The feasibility of using a concentration-based diffusion coefficient to populate intermediate pressure steps instead of the standard time-lag diffusion will be explored. This method can be used in complement with the time-lag diffusion to calculate diffusion coefficients at secondary-step pressures, since the time-lag method accounts for the first arrival concentration and cannot directly yield diffusion coefficients of non-first arrival pressures. 

This is strictly due to the boundary conditions involved in the Fickian diffusion model, which is applicable to slow, uniform, one-dimensional diffusion of a permeant through a membrane that separates donor and acceptor compartments. The transport equation of such a model can be described as [43]:

(7) $$\frac{\partial c}{\partial t}=D\frac{\partial^{2}c}{\partial x^{2}}$$

where *C* is permeation concentration; *D* is the diffusion coefficient of the permeant at infinite dilution; and *t* and x are the temporal and special variables, respectively. This is Fick’s second law and is subjected to the following boundary conditions:

$$t\leq 0,\;\;x=0\;\;\;\;c=c_{0}$$

t≤0,  x>0    c=0

$$t>0,\;\;x=0\;\;\;\;c=c_{0}$$

t>0,  x=l    c=0

in which the infinite donor source of the permeant is located at *x* = 0 and the membrane has a thickness of *l*. 

Existing equations that are standard in the literature for calculating $D_{i}$ from permeation experiments use a form of the time-lag method introduced in Equation (6). Variations of the time-lag model such as the Yen and Shih model [44], Laplace and Fourier analytical methods as demonstrated by McBreen et al. [45], and others [46,47,48] normally introduce a dimensionless time variable, τ, to characterize a percentage (e.g., $Q_{i}/Q_{\infty }=$ 0.63, 0.65, 0.83, etc.) of the equilibrium flux as sweet spot for determining $D_{i}$. However, all methods assume first arrival pressures that consider a *c* = 0 scenario by default. As a result, the existing time-lag form of models cannot accurately predict $D_{i}$ at secondary arrival pressures. The scope of the current study does not include further evaluation of diffusion models, but only presents the reasoning for the resort to concentration-based $D_{i}$ in aging analysis.

Concentration-based $\;D_{i}$, on the other hand, can be determined relatively easily by equating the gradient of Equation (5) with the slope calculated from the accumulated volume versus time data, recorded by the gas chromatographer. Rearranging for concentration gives:

(8) $$C_{i}=\frac{\nabla Q_{acc,i}\cdot l}{A\cdot D_{i}}$$

Note that in Equation (8) the gradient of the accumulated volume is divided by the inlet surface area, *A*, to establish one-dimensional diffusion and obtain a dimensionless concentration behavior. 

Once a trend of concentration values is obtained at various pressure steps, a best-fit estimate of the concentration curve determined from the first arrival $D_{i}$ values using Equation (8), $C_{i,est\;}$, can be used to recalculate an estimate of interpolated diffusion coefficients.

(9) $$D_{i,est}=\frac{\nabla Q_{acc,i}\cdot l}{A\cdot C_{i,est}}$$

This method is particularly useful when the monitoring of within-film concentrations at different conditions is experimentally challenging. 

### 3.4. Fractional Free Volume

The amorphous region of a polymer can be rearranged as the temperature is increased above the glass transition temperature, $T_{g}$. As a result, the fractional volume of voids in the amorphous region increases linearly with temperatures above $T_{g}$ [9,49,50,51].

It naturally follows then that it is of interest to investigate whether changes in fractional free volume as a result of aging can be determined through long-term permeation tests that involve pressure sweeps. The diffusion coefficient, $D_{i}$, can be correlated to the fractional free volume, FFV, by Doolittle’s equation of the form [9,16]

(10) $$D_{i}=A_{0}\;exp\left(-\frac{B}{FFV}\right)\;$$

where $A_{0}$ and *B* are empirical constants and *FFV* can be described as

(11) $$FFV=\frac{V_{f}}{V_{sp}}\;$$

where $V_{f}=V_{sp}-1.3V_{vw}$ and $V_{vw}$ of PE is 20.56 cc mol^−1^ [52].

Therefore, monitoring ln(D) at different pressures enables the possibility of calculating changes in 1/FFV; consequentially, alterations in the mechanical properties of polymeric materials can be inferred.

Decreasing gas sorption trends with higher pressures were reported in the literature [24,53,54,55,56,57], where the behavior was assigned to a decrease in the polymers’ fractional free volume at these conditions. Fujiwara et al. [58] reported a decreasing behavior of permeability, diffusion, and sorption coefficients of various types of PE specimen ranging from low to high densities with 1/FFV.

A relationship between $K_{i}$ and FFV of the same form of Equation (10) exists as

(12) $$K_{i}=A_{0}\;exp\left(-\frac{B}{FFV}\right)\;$$

which can be especially beneficial when the permeation experiment includes various pressure steps. This is because $K_{i}$ is relatively easier to determine at secondary pressure steps, using Equation (1), than $D_{i}$.

### 3.5. Fugacity Coefficient 

The gas feed pressure that the polymer membrane is exposed to can increase or, in some cases, decrease permeability [7,15,27,36,59]. Therefore, the rate of permeation is not only dependent on the polymeric structure, but also dependent on the surrounding pressure and temperature to which the polymer is exposed.

The continuous flow method allows one to study the permeation of gas mixtures through a membrane. Different gases existing in a single feed pressure exert individual partial pressures corresponding to the component gases. However, the current study explores the added value of using fugacity instead of feed pressure in permeability coefficient calculations when there is only a single supercritical gas in action. 

The fugacity coefficient ϕ can be described as [60]

(13) $$\ln\left(\phi_{i}\right)=\int_{0}^{P}\left(\frac{P{\bar{V}}_{i}}{RT}-1\right)\frac{dP}{P}\;\;$$

where *P* is the feed pressure, ${\bar{V}}_{i}$ is species *i* molar volume, *R* is the ideal gas constant, and *T* is absolute temperature. Fugacity of species i, then, is related to feed pressure by

(14) $$f_{i}=\phi_{i}y_{i}P,\;\mathrm{where}\;0\leq \phi \leq 1\;$$

Note that the fugacity term is always less than the ideal partial pressure, $y_{i}P$, value because the fugacity coefficient does not exceed 1.

### 3.6. Activation Energies of Transport Coefficients

For PE-based materials, it is expected that the value of $K_{i}$ will change exponentially as the temperature increases [9]:

(15) $$K_{i}=K_{0}\exp\left(-\frac{E_{aK}}{RT}\right)$$

where $K_{0}$ is constant, $E_{ak}$ (kJ mol^−1^) is the activation energy for permeation to occur, R (kJ mol^−1^ K^−1^) is the ideal gas constant, and T (K) is the absolute temperature [15,61].

## 4. Results

**Figure 2:**
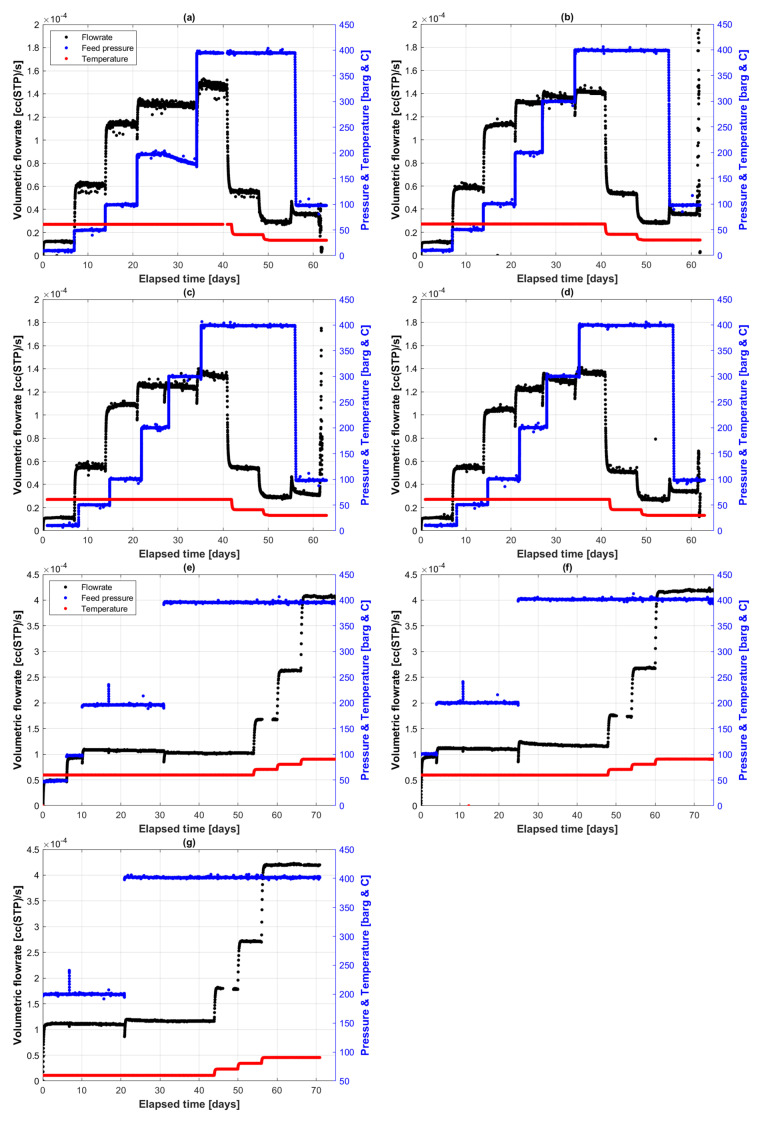
Volumetric flowrates of CO_2_ permeant to 2mm PE-RT film at various pressure and temperature steps, detected by a GC through constant N_2_ sweep. (**a**–**d**) First permeation round; (**e**–**g**) second permeation round. Black is flowrate, blue is pressure, and red is temperature.

Figure 2 shows the permeation profiles of the two permeation runs. In the first run (Figure 2a–d), four identical cells of PE-RT exposed to supercritical CO_2_ started from 10 barg and went up to 400 barg, while the temperature started from 60 °C and was gradually reduced to 30 °C. In the second run, a similar pressure profile to run 1 was used, but the starting pressure was changed to allow high pressures to be first arrivals. Hence, the starting pressure was 50 barg in cell 2A (Figure 2e), 100 barg in cell 2C (Figure 2f), and 200 barg in cell 2B (Figure 2g), all of which went stepwise to 400 barg. The temperatures in the second round were ascending as opposed to the first round, also starting at 60 °C, but going to 90 °C in 10 °C increments.

**Figure 3:**
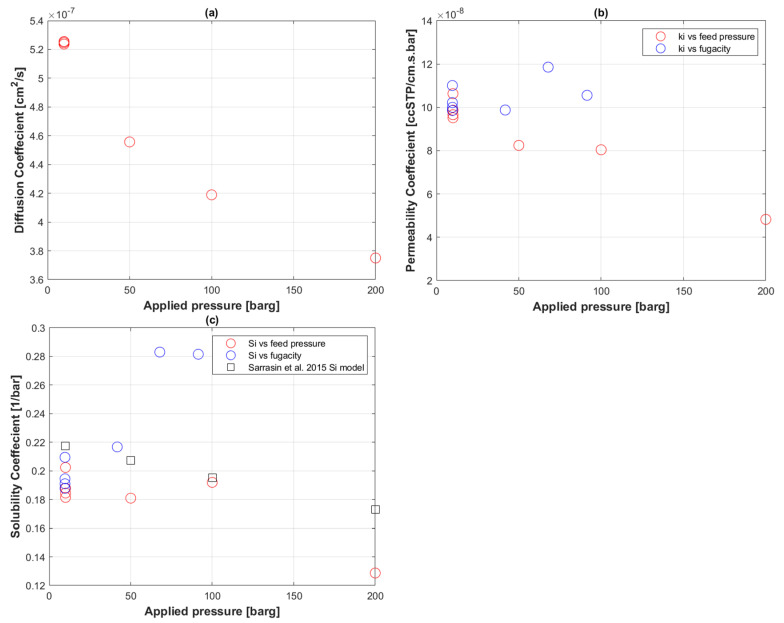
Transport coefficients of CO_2_ permeation to PE-RT at 60 °C of first arrival pressure steps. (**a**) Diffusion coefficient; (**b**) permeability coefficient; (**c**) solubility coefficient [26].

Figure 3 shows the transport coefficients summarizing all the permeation runs of PE-RT, where the diffusion coefficient was calculated using the standard time-lag method (Figure 3a) and the permeability coefficient was determined from the classical solution-diffusion model (Figure 3b). Figure 3c compares the calculated solubility coefficients to a solubility prediction by Sarrasin et al. [26] based on applied pressure.

**Figure 4:**
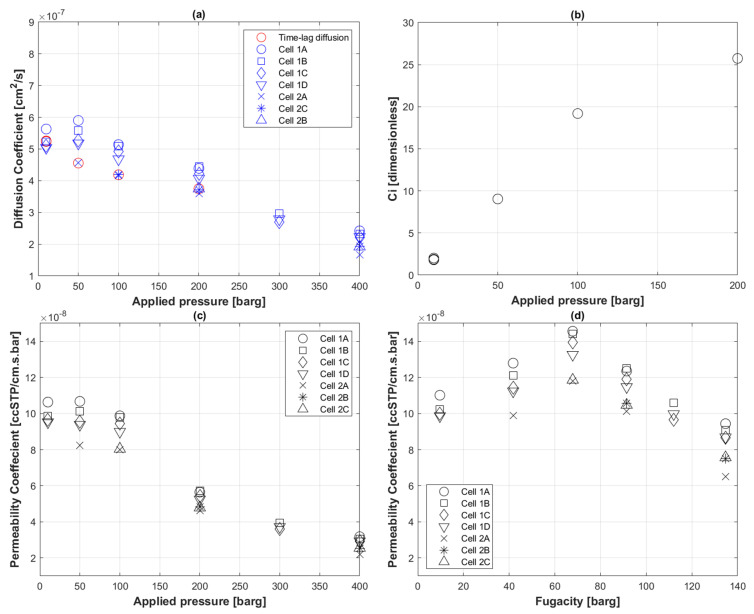
Transport coefficients of CO_2_ permeation to PE-RT at 60 °C at all pressure steps. (**a**) Diffusion coefficient; (**b**) calculated within-film concentrations from first arrival $D_{i}$; (**c**) feed pressure-based permeability coefficient; (**d**) fugacity-based permeability coefficient.

Figure 4a portrays the attempt of populating the diffusion coefficient of higher-pressure steps using the slope-concentration Equation (9) explained earlier. The best-fit equation of first arrival concentration values plotted in Figure 4b was a logarithmic fit with an *R*-value of 96%.

(16) $$C_{i,est}=6.9445\ln\left(P\right)-14.187\;\mathrm{with}\;R=0.9612$$

Figure 4c shows permeability coefficients of all pressure steps in the experiment using feed pressure, and Figure 4d show the same permeability coefficients calculated with fugacity coefficients instead of ΔP in Equation (1).

**Figure 5:**
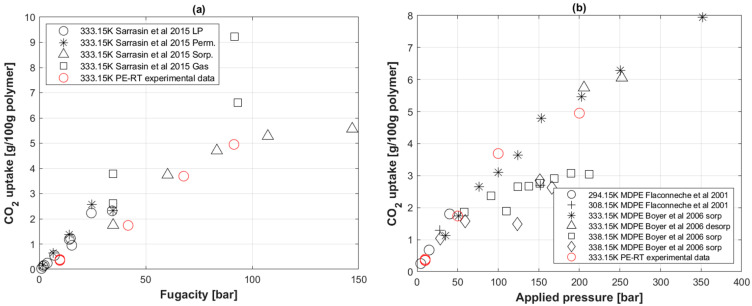
(**a**) CO_2_ uptake of PE-RT compared to HDPE; (◯) and (*) are from low and higher permeation experiments, respectively; (∆) is from sorption cell coupled with an infrared detector; (□) is from direct gas uptake measurements [26]. (**b**) CO_2_ uptake of PE-RT compared to MDPE [25,62].

Figure 5a shows a comparison of CO_2_ uptake concentration in grams of gas per 100 g of PE-RT specimen to what was experimentally reported by Sarrasin et al. [26] with HDPE. The same data were then compared to those of Flaconneche et al. [62] and Boyer et al. [25] for CO_2_ uptake with medium-density polyethylene (MDPE) at similar temperatures. 

It is worth noting that CO_2_ uptake in polymers was calculated using the concentration values obtained from Equation (8) and then divided by gas and polymer densities at standard temperature and pressure conditions.

(17) $$CO_{2}\;uptake=\frac{C_{i}\;\left[ccSTP/cc\right]\;}{\rho_{CO2,\;STP}\rho_{PERT,\;STP}}\times 100\;[g\;\mathrm{gas}/100g\;\mathrm{polymer}]\;$$

**Figure 6:**
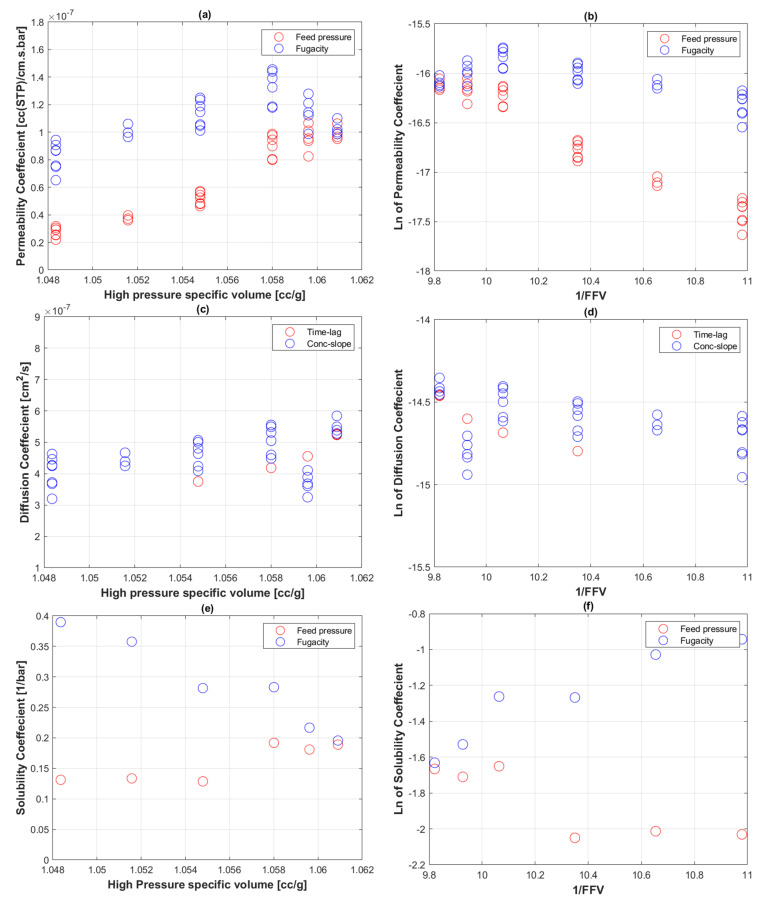
Transport coefficients of CO_2_ to PE-RT at 60 °C plotted with specific volume and 1/FFV. (**a**,**b**) Permeability coefficient; (**c**,**d**) diffusion coefficient; (**e**,**f**) solubility coefficient.

Figure 6 displays the profile of transport coefficients (permeability, diffusion, and solubility) against specific volume at high pressures and 1/*FFV*. The fractional free volumes presented were calculated from interpolations of published PE specific volume $V_{sp}$ values at 0 MPa and 50 MPa at ranging temperatures [63]. Ideally, however, fractional free volumes of the specific PE-RT grade coming from PVT analysis should be used.

**Figure 7:**
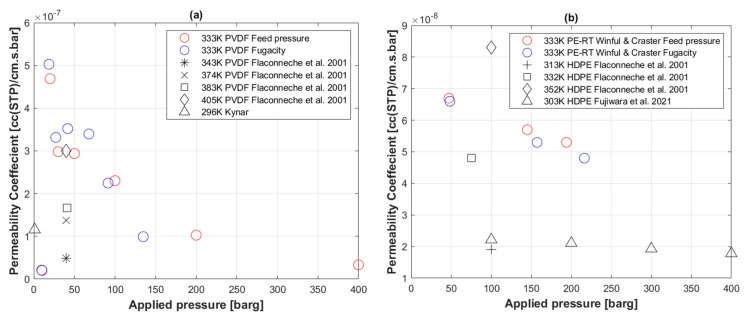
(**a**) Permeability coefficient of CO_2_ to PVDF compared to literature data [62]; (**b**) permeability coefficient of H_2_ to PE-RT compared to literature [58,62,64].

Figure 7a shows permeability coefficient profiles against pressure of a similar geometry PVDF specimen with supercritical CO_2_. Figure 7b shows a PE-RT specimen exposed to H_2_, as conducted by Winful and Craster [64]. Both experiments were carried out at 60 °C.

**Figure 8:**
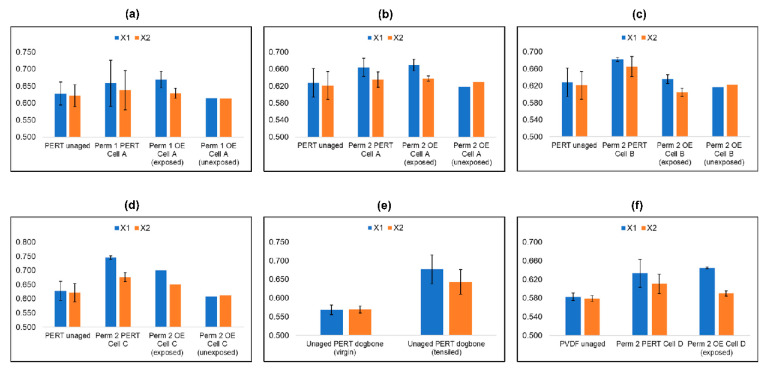
DSC profiles of 2mm polymer discs exposed to different conditions of induced aging. (**a**) Cell 1A PE-RT exposed to up to 60 °C and 400 barg starting from 10 barg; (**b**) cell 2A PE-RT exposed to up to 90 °C and 400 barg starting from 50 barg; (**c**) cell 2B PE-RT exposed to up to 90 °C and 400 barg starting from 200 barg; (**d**) cell 2C PE-RT exposed to up to 90 °C and 400 barg starting from 100 barg; (**e**) comparison of virgin and tensiled PE-RT at STP until necking; (**f**) cell 2D PVDF exposed to up to 90 °C starting from 10 barg. *Nomenclature:* X1 (blue) is 1st crystallinity cycle; X2 (orange) is 2nd crystallinity cycle; Perm 1 is 1st permeation round at conditions up to 60 °C and 400 barg; Perm 2 is 2nd permeation round at conditions up to 90 °C and 400 barg; OE (exposed) means part of aged specimen exposed to thermal loading but not to pressure loading; OE (unexposed) means part of the aged specimen not exposed to thermal or pressure loadings.

Figure 8 depicts the DSC profile of a series of PE-RT specimens exposed to different conditions of induced aging, including the first permeation round where the highest thermal and pressure loads were 60 °C and 400 barg (Figure 8a), the second permeation round with 90 °C and 400 barg as the highest loads (Figure 8b–d), tensile tests of a dog-bone PE-RT specimen until necking was achieved (Figure 8e), and lastly a PVDF specimen exposed to similar conditions to the PE-RT in the second permeation round (Figure 8e). 

The outermost left columns in Figure 8a–d show a representative unaged PE-RT sample, the second column shows a specimen taken from the center of the disc where direct contact with supercritical CO_2_ occurred, the third column shows a specimen taken from the side where only thermal fatigue was experienced but not pressure loading, and lastly the far-right column includes a part of the same disc that was covered and was not in direct contact with pressure or heat throughout. 

Polymer percent crystallinity, *X*, is calculated as the ratio between the experimentally obtained enthalpy of heat and the assumed enthalpy of a 100% crystalline specimen. A value of 293 J/g was used as the enthalpy of a 100% crystalline PE [65,66], and 104.5 J/g was used for PVDF [67].

(18) X=ΔH/Xc ,

where $X_{c,PE}=293\frac{J}{g}$ and $X_{c,PVDF}=104.5\frac{J}{g}$.

**Figure 9:**
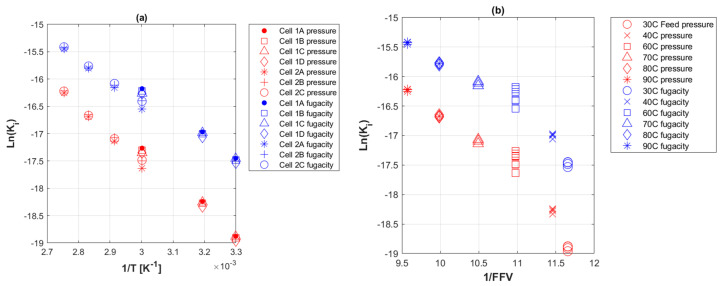
Arrhenius type plots of the permeability coefficient of CO_2_ to PE-RT permeation cells. (**a**) Natural log of permeability coefficient against 1/T; (**b**) natural log of permeability coefficient against 1/FFV.

The effect of temperature on the permeability coefficient could be assessed by an Arrhenius type plot of Equation (15), i.e., $\ln\left(K_{i}\right)$ versus 1/T as depicted in Figure 9a. Another evaluation of how temperature affects FFV can be described by plotting the Doolittle relationship of Equation (12), as shown in Figure 9b.

## 5. Discussion

### 5.1. Continuous Flow Permeation Volumetric Fluxes

#### 5.1.1. First Permeation Round

The first permeation round (Figure 2a–d) had identical conditions, starting from 10 barg and 60 °C. It can be seen that the flowrate increased proportionally with pressure, at constant temperature, with a temporary pressure hysteresis effect at the pressure changing moments. This is explainable by the length of the nitrogen-filled conduit that carries the permeated concentration front wave to the GC. 

The flowrates across the four identical cells were very similar, averaging around 1.4 × 10^−4^ cc(STP) s^−1^, at constant 400 barg and 60 °C. The effect of temperature on the flowrate can be seen starting from 42 days, when the temperature was dropped from 60 °C to 40 °C, and the flowrate decreased as a result to less than half the value of about 0.55 × 10^−4^ cc(STP) s^−1^. The trend continued with another drop in temperature from 40 °C to 30 °C, where the flowrate almost halved again to 0.3 × 10^−4^ cc(STP) s^−1^. This indicates that the PE-RT is significantly more susceptible to critical CO_2_ permeation at higher temperatures.

#### 5.1.2. Second Permeation Round

The purpose of the second round of permeation experiments was to conduct the same experiments of round 1 with a virgin PE-RT specimen, but by starting with the higher-pressure steps, with the aim of determining transport coefficient changes at those higher pressures. The starting pressures in cells 2A (Figure 2e), 2C (Figure 2f) and 2B (Figure 2g) were 50, 100 and 200 barg, respectively.

By taking the equilibrium condition at 400 barg and 60 °C as common grounds for comparison to the fluxes in the first permeation round, we see that flowrates at this condition are now slightly lower, averaging around 1.2 × 10^−4^ cc(STP) s^−1^. In cell 2A (Figure 2e), it is seen that the flowrate did not change when the pressure was increased from 200 to 400 barg, although the pressure hysteresis effect was present. 

The effect of temperature was explored in ascending steps this time, and the result was similar, but in reverse, to what was seen in the first permeation round. At constant 400 barg, the temperature increase from 60 °C to 70 °C resulted in a flowrate rise of about 45%. Similarly, going from 70 °C to 80 °C and from 80 °C to 90 °C increased the detected CO_2_ at the permeation interface by 58% and 55%, respectively. 

One can note that the effect of high temperature on CO_2_ permeation through PE-RT is higher than that of higher pressure. In fact, the effect of increasing pressure seems diminishing, whereas the effect of increasing temperature seems linear. Therefore, although the PE-RT melting point averages around 132 °C, the operating envelope of PE-RT-based specimens in supercritical CO_2_ environments should be limited to temperatures conservatively below 90.

The flowrate at different pressure steps when compared between the first and second permeation rounds seems to be always lower when the first arrival pressure is higher. For instance, flowrates at 200 barg in cells 2A–C (Figure 2e–g) are always lower than those in cells 1A–D (Figure 2a–d), and the trend stands true. This is indicative of a negative slope diffusion behavior within polymer film, where it decreases with increasing arrival fluid pressure.

### 5.2. Transport Coefficients of CO_2_ to PE-RT

The diffusion coefficients, $D_{i}$, in Figure 3a were calculated using the first arrival pressure steps via the time-lag Equation (6). This calculation allowed for seven data points only, four from the first permeation round and three from the second. All four points from the first round started with 10 barg and thus clustered around 5.25 × 10^−7^ cm^2^ s^−1^. There is an obvious decreasing trend of $D_{i}$ with increasing pressure. It should be noted, however, that $D_{i}$ is sensitive to noise from various experimental parameters, and abrupt peaks of volumetric flux had to be cleaned first. 

The permeability coefficients, $K_{i}$, of the first arrival pressure points were determined by Equation (1), and their decreasing linear behavior is depicted in Figure 3b. In the same plot, fugacity values of pure CO_2_ at the corresponding PVT conditions, determined by Refprop NIST ® software using the Peng–Robinson equation of state [68], when used in place of feed pressure in Equation (1), did not yield a similar linear behavior. 

Solubility coefficients, $S_{i}$, on the other hand, were calculated from the ratio of $K_{i}$ to $D_{i}$ in Equation (2) using feed pressure and pure CO_2_ fugacity values as shown in Figure 3c. Using feed pressures for Equation (4), the sorption isotherm model proposed by Sarassin et al. [26] was plotted by assuming the same assumptions as [26] of no plasticization effect, $K_{Di}=$ 0.22 cm^3^ cm^−3^ bar^−1^, and $\beta_{CO_{2}}=-$0.0012 bar^−1^. The model predicted a linear decrease in $S_{i}$ with the increasing feed pressure, partly because Henry’s law was assumed to hold in the background. Even if affected by noise, the $S_{i}$ calculated from Equation (2) and feed pressure is not far off from the model’s prediction. The effect of assuming σ=0 in Equation (4) was masked by the error margin of $S_{i}$, hence this method has not yet proved effective to monitor aging. Fugacity-based $S_{i}$, in contrast, did not show a readable trend with the given seven data points.

Equations (8) and (12) were used to populate the diffusion coefficient values of the various pressure steps by determining $C_{i}$ of CO_2_ within the polymer film from the time-lag $D_{i}$ and then back calculating $D_{i}$ using the best-fit Equation (12) of $C_{i}$ plotted against various pressure steps (Figure 4b). Upon doing so, a decreasing overall trend of the diffusion coefficient with increasing feed pressure appeared, as noted in Figure 4a. This is especially true when feed pressure exceeds 73.8 barg, which is when CO_2_ becomes supercritical. Note that all plots in Figure 4 were at 60 °C. The $D_{i}$ behavior of supercritical CO_2_ to PE-RT is comparable in trend but 50% lower than what has been reported by Fujiwara et al. [58] on H_2_ diffusion to different PE grades, especially HDPE and HDPE(PE-100).

$K_{i}$, unlike $D_{i}$ using time lag, does not depend on the history of the pressure profile; hence, by using the $Q_{max,i}$ of the intermediate pressure steps, one can determine their corresponding $K_{i}$ values through Equation (1). It is worth noting that the surface area, *A*, of the sinter where the polymer is exposed to the feed pressure, and polymer thickness, *l*, were assumed constant throughout the permeation process. Figure 4c shows the inversely proportional $K_{i}$ with applied pressure, which is compliant with the assumptions of classical permeation (Equation (1)) and in agreement with the findings in the literature on the permeation of CO_2_ through HDPE membranes [24,62,69,70,71]. 

The same process was used to calculate fugacity-based $K_{i}$ shown in Figure 4d. The effect of CO_2_ fugacity on $K_{i}$ can be described as a two-stage phenomenon. At fugacity values less than 73.8 barg, $K_{i}$ is increasing with pressure, and this may be attributed to temporary polymer swelling that has possibly masked the fugacity-based $K_{i}$ linearity when using only first arrival pressures (Figure 3b). At fugacity values higher than 73.8 barg, a similar trend to the feed pressure-based $K_{i}$ was observed, although considerably higher. This suggests that the use of fugacity for data-rich permeation analysis could magnify transitional $K_{i}$ behavior to allow inference of temporary structural changes. However, one could also argue that $K_{i}$ at lower pressures before reaching supercritical CO_2_ did not significantly change with feed pressure (Figure 4c).

### 5.3. CO_2_ Uptake in PE-RT

The permeation data of CO_2_ uptake in PE-RT at 60 °C are in agreement with the sorption data of HDPE of PE-80 grade presented by Sarassin et al. [26] (Figure 5a). It is worth noting that both the PE-RT and PE-80 data show asymptotic behaviors at higher fugacity values (more obvious for PE-RT in Figure 4b), suggesting a deviation from Henry’s law of constant sorption (Equation (3)). Nevertheless, Henry’s law was assumed in transport coefficients’ calculations. The diminishing slope effect can be attributed to Langmuir-type sorption. Langmuir-mode sorption occurs when polymer–penetrant interactions dominate, in which case the penetrant molecules take place in the microvoids of the specimen provisionally at higher pressures [72].

Comparing the CO_2_ uptake in MDPE found in the literature [25,62] to PE-RT (Figure 5b) at similar temperatures shows small differences. This topic requires further study as the former may have a higher fractional free volume due to its packing density being lower [25].

### 5.4. Fractional free Volume of PE-RT

The gradient of the fugacity-based $K_{i}$ plotted against specific volumes of PE-RT (Figure 6a) shows agreement with that of feed pressure-based $K_{i}$ at high pressures until $V_{sp}=$ 1.058 cc g^−1^, even though the fugacity-based values are noticeably higher. This trend is expected because higher fugacity values are required to achieve the same effects as feed pressure given that the fugacity coefficient ϕ is less than 1. At lower pressures, represented by specific volumes higher than 1.058 cc g^−1^ (equivalent to 100 barg), the behavior of fugacity and feed pressure permeability coefficients is opposite. 

$\ln\left(K_{i}\right)$ appeared to decrease with increasing 1/FFV, and the fugacity-based values are also higher, but with a lower negative slope (Figure 6b). Overall, the positive slope trend of $K_{i}$ against $V_{sp}$ and the negative slope of $\ln\left(K_{i}\right)$ against 1/FFV are comparable to Fujiwara’s [58] data of H_2_ permeation to HDPE. 

The diffusion coefficient, $D_{i}$, increased with specific volume (Figure 6c) and decreased with increasing 1/FFV (Figure 6d), which is in agreement with the prediction of Equation (10). Similar to the trend of $D_{i}$, the solubility coefficient $S_{i}$ increased with specific volume (Figure 6e) and decreased with increasing 1/FFV (Figure 6f). However, the trends were more exaggerated, especially in distinguishing fugacity and feed pressure-based solubility coefficients.

Lastly, it is worth noting that the extent of change observed in the FFV values of PE, from literature PVT interpolations, at 60 °C and pressures from 10 barg to 400 barg was within 1.2% (Figure 6b,d,f). The change with temperature (30 °C to 90 °C), at constant 400 barg, was within 2.0% (Figure 9b). The extent of the FFV change, which is relatively small, can be significantly reflected in the specimens’ densities under those conditions [73,74]. An ultrasound sensor can possibly pick up such structural alteration through the inverse calculation of elastic moduli [75]. This notion will be explored further in a subsequent study focusing on the feasibility of bespoke ultrasonic transducers for measuring polymeric structural alterations non-destructively. 

### 5.5. Transport Coefficients of CO_2_ to PVDF

The permeability coefficient of CO_2_ with PVDF in Figure 7a appeared to decrease with increasing pressure, which is similar to the behavior of PE-RT, although about a magnitude higher. Fugacity-based $K_{i}$ with PVDF did not show significant differences to feed pressure-based $K_{i}$. However, one can argue about the change in slope at the supercritical pressure boundary (73.8 barg interface). Nonetheless, that discontinuity is still less apparent in comparison to PE-RT (Figure 4c,d). 

Flaconneche et al. [62] conducted variable temperature permeation tests at isobaric conditions of about 40 barg, although seemingly for a different grade of PVDF to the one used in this study, and showed the effect of increasing temperature on $K_{i}$. As the temperature increased from 70 °C to 132 °C, $K_{i}$ appeared to increase proportionally. 

In Figure 7a, the $K_{i}$ of Kynar—a conventional grade of PVDF from Arkema [76]—at standard temperature and pressure conditions is also shown for reference.

### 5.6. Transport Coefficients of H_2_ to PE-RT

The permeability of H_2_ with PE-RT appeared to also decrease with increasing pressure, as depicted in Figure 7b. However, there seems to be no difference between feed pressure- and fugacity-based $K_{i}$ behaviors for H_2_ with respect to PE-RT. This suggests that the fugacity effect depicted for CO_2_ with PE-RT in Figure 4c,d is real. Since H_2_ does not have a supercritical phase, it is apparent that the change in $K_{i}$ behavior displayed by fugacity-based calculations is controlled by a gas’s physical characteristics when undergoing a supercritical phase. 

Flaconneche et al. [62] conducted variable temperature permeation tests of H_2_ to HDPE, which could be seen to greatly affect $K_{i}$ by increasing it proportionally with temperature. Fujiwara et al. [58] carried out a similar series of permeation tests at high pressures and 30 °C and showed $K_{i}$ to decrease with increasing pressure (Figure 7b). Their used temperature is lower than the 60 °C condition used in this study, and as a result, their $K_{i}$ values are lower, which suggests that the permeation behavior of the PE-RT specimen used in this study is in line with that of HDPE specimen used by Fujiwara et al. 

Even if the comparison was made with only three data points, the general trend seems to be that the permeability of H_2_ to PE-RT (Figure 7b) is slightly lower than the permeability of CO_2_ to PE-RT (Figure 4c,d). 

### 5.7. Differential Scanning Calometry of Aged Specimen

Comparing the unaged and aged PE-RT specimens from the first permeation round shows an increase in the first DSC cycle percent crystallinity, $X_{1}$, where the sample was in direct exposure (column 2 in Figure 8a) or radial exposure (column 3 in Figure 8a) to supercritical CO_2_. The increase in $X_{1}$ in both scenarios was similar, suggesting equivalent amounts of thermal and pressure loads. 

The first and last columns of Figure 8a gave similar $X_{1}$ values, suggesting that the outermost areas of the disc that underwent permeation (column 4 Figure 8a) were not exposed to thermal or pressure loads at all. 

Results from the second permeation round of PE-RT (Figure 8b–d), where the specimens were exposed to similar pressure steps but higher thermal loads, showed an even higher increase in $X_{1}$ for the direct (second column) and radial (third column) exposure scenarios. The first and fourth columns showed similar, unchanged $X_{1}$ values, representing a control group. 

The crystallinities of the second cycle with DSC usually are the same because the second heat cycle is meant to obliterate the thermal history of the specimen. Thus, the increases in X observed due to permeation experiments were not permanent and were erased once chain reallocation took place. However, this was not true for cells 2B and 2C (second column Figure 8c,d), where the starting pressures were 200 barg and 100 barg, respectively, because the $X_{2}$ values did not return to their original values. This suggests permanent aging effects. Moreover, it shows that initial high-pressure fatigue can have a more permeant aging effect on PE-RT than starting with low pressure and then gradually increasing.

To evaluate the induced aging of the permeation experiments on the PE-RT specimen and put it into perspective, mechanical tests were carried out on unaged PE-RT dog-bones, which showed a more dramatic increase in $X_{1}$ when compared to the permeation experiment loads. In addition, the necking of the dog-bones is vividly translated by the increase in $X_{2}$ in Figure 8e. 

The PVDF specimen when exposed to supercritical CO_2_ resulted in further chain mobility than PE-RT (Figure 8f) if the extent of the increase in $X_{1}$ could be described as such. The specimen that was in direct exposure to supercritical CO_2_ (2^+^ column in Figure 8f) showed a similar increase in $X_{1}$ to the one exposed radially (third column in Figure 8f). However, its higher $X_{2}$ suggests that the directly exposed areas were more permanently affected.

### 5.8. Permeation Coefficient with Temperature

The relationship portrayed in Figure 9a of $\ln\left(K_{i}\right)$ with 1/T shows a constant slope that agrees with the estimation of Equation (14) and the expectation for PE-based materials $K_{i}$ to change exponentially with temperature [9]. The plot also shows fugacity-based values of $\ln\left(K_{i}\right)$, in line with the earlier explanation, to be higher than feed pressure-based values at the same temperatures. The calculated values of $E_{ak}$ and $k_{0}$ as a result from Equation (14), were 39.46 kJ mol^−1^ and 0.041, respectively. This is comparable to the reported apparent activation energy of CO_2_ through HDPE by Zhang et al. [77] as 32.61 kJ mol^−1^, as well as to CO_2_ in PE described by Michaels and Bixler [78] to range between 30 and 39 kJ mol^−1^. 

Furthermore, a similar trend was depicted between $\ln\left(K_{i}\right)$ and 1/FFV at isobaric 400 barg and changing temperatures (Figure 9b), where the slope was constant and fugacity-based values were higher than those of feed pressure. 

## 6. Conclusions

This study has included a spectrum of experiments, including CO_2_ permeation at elevated temperatures and pressures through PE-RT and PVDF, H_2_ permeation through PE-RT; and DSC tests on virgin and permeated specimens. The findings summarize the aging attributes of those polymers upon exposure to plasticizing gases through the analysis of their transport coefficients. It was shown that the diffusion and permeation coefficients of supercritical CO_2_ to PE-RT agreed with the equations of Fickian diffusion and the classical permeation model, where the diffusion and permeation coefficients decreased in general with increasing feed pressure. The response of PE-RT transport coefficients to temperature was higher than pressure. Therefore, the operating envelope of PE-RT specimens in supercritical CO_2_ environments should be limited to conservative temperatures considerably below 90 °C.

The time-lag method using the first arrival diffusion coefficient is a consistent approach but sensitive to uncertainty in the measurement of experimental parameters; thus, populating it with a concentration-based diffusion coefficient complements the dataset. 

While PE-RT sorption of CO_2_ fitted roughly with the model predicted by Sarassin et al., assuming no plasticization, CO_2_ uptake in PE-RT was more comparable to what has been reported for HDPE, and much lower than that of MDPE at similar pressure and temperature conditions.

Fugacity amplifies transitional trends, such as the interface between gaseous and supercritical CO_2_ that is less visible with a feed pressure-based permeability coefficient. The fugacity effect seems real when compared between CO_2_ to PE-RT and H_2_ to PE-RT, where there was no discontinuity of $K_{i}$ with H_2_ at the same pressure steps, because H_2_ does not have a supercritical phase. Furthermore, $K_{i}$ values reported in the literature for H_2_ at 30 °C with HDPE were relatively higher than what was determined for PE-RT at 60 °C, suggesting the advantage of PE-RT over HDPE for H_2_ permeation. The $\ln\left(D_{i}\right)$ versus 1/FFV trend agreed with Doolittle’s equation, and $\ln\left(K_{i}\right)$ versus 1/FFV showed linear behavior both at isobaric conditions of variable temperature and isothermal conditions of variable pressure. The activation energy of permeation showed an agreement with what has been reported for PE. The extent of change in FFV in PE at changing temperature was higher than that of changing pressure, which is due to the greater susceptibility of PE-RT to CO_2_ permeation at high temperatures than at high pressures. 

Lastly, DSC experiments showed that the crystallinity of PE-RT after exposure to supercritical CO_2_ at 90 °C was the highest. In addition, an initial high-pressure CO_2_ flux displayed more permeant aging effects on PE-RT than lower starting pressures, as indicated by the increased crystallinities. Other techniques, such as ultrasonic assessment proposed in an ongoing study, may also leverage the findings of this study. 

## Data Availability

Not applicable.
